# Evaluation of a Porcine Dermal Collagen (Permacol) Implant for Abdominal Wall Reconstruction in a Pediatric Multitrauma Patient

**DOI:** 10.1155/2014/585723

**Published:** 2014-03-10

**Authors:** Idit Melnik, Youri Mnouskin, Edna Verdiger Kurzbart, Boris Yoffe

**Affiliations:** ^1^Department of General and Vascular Surgery, Barzilai Medical Center, Hahistadrout Street 2, 78278 Ashkelon, Israel; ^2^Department of Pediatric Surgery, Barzilai Medical Center, Hahistadrout Street 2, 78278 Ashkelon, Israel

## Abstract

The presence of a contaminated surgical field in abdominal wall defects caused by trauma presents a challenge for surgeons. Both primary suture and synthetic meshes are strongly discouraged as surgical treatments in such cases. We describe the use of a porcine dermal collagen (Permacol) implant in an eight-year-old patient with multiple injuries. Three months after discharge, the child remains well with good cosmetic results. He is free of pain and has returned to full activity levels with complete wound closure and without any evidence of residual hernia. In conclusion, our experience indicates that the use of Permacol can be considered an efficient technique for reconstructing an infected abdominal wall defect of a pediatric multitrauma patient.

## 1. Introduction

Abdominal wall defects caused by trauma, incisional hernias, or tumor resection present a formidable challenge for surgeons, as the size of such defects and the presence of contamination of the surgical field can complicate the surgical procedure. The treatment of small incisional hernias commonly consists of simply suturing opposing fascial edges. However, as the defect increases in size, recurrence rates may rise by up to 50% [1–4]. Thus, in cases of large defects, most surgeons agree that the defect should be repaired in a tension free manner using a prosthetic mesh material [5]. Although these meshes increase abdominal wall strength [6], they are associated with serious complications such as fistula formation, adhesions, skin erosion, and increased susceptibility to infections [7]. Therefore, the use of synthetic meshes in contaminated fields, which are common in trauma, has high rates of morbidity and is strongly discouraged [8]. Recently, the focus on treatment has shifted toward bioprosthetic meshes that provide strength and promote host tissue incorporation and infection resistance, all of which combine to make them particularly well suited to use in contaminated fields. Permacol is a relatively new acellular porcine dermal matrix for use in abdominal wall reconstruction. Structurally similar to the human dermis, it is purported to be equally nonallergenic and nontoxic. In addition, the chance of suffering a foreign body reaction is much lower with Permacol than with conventional prosthetic meshes. We present here our case experience with a Permacol mesh in the reconstruction of the abdominal wall of an eight-year-old patient suffering from multiple injuries.

## 2. Case Report

This case involves an eight-year-old boy from a Palestinian refugee camp who was badly wounded during an explosion from which he suffered multiorgan shrapnel injuries. According to the transfer letter, he was evacuated to a nearby hospital in Gaza where he underwent a right hemicolectomy due to direct shrapnel injury to the colon and an internal fixation of a fracture in the left ilium. He also suffered from injuries in his left hand and underwent amputation of the first finger. With the help of a humanitarian organization, a day after his injury, he was transferred across the border for additional treatment to our center.

The patient presented to the emergency room unconscious and intubated but stable. A brief physical exam of the abdomen revealed diffuse tenderness and peritoneal signs with a midline laparotomy scar. There was a shrapnel entry wound in the left abdomen and an exit wound with massive lack of skin in the left groin. He underwent total body CT that demonstrated mild cerebral edema and mild right hydropneumothorax with a fracture in the fourth rib. In the abdomen-bowel, thickening due to hypoperfusion and a crushed fracture with fragment movement in the left iliac bone that was under fixation were observed. A right chest tube was inserted in the patient for drainage of air and blood leaks. Blood tests revealed HGB: 7.75 and WBC: 13.42 K with metabolic acidosis (HCO_3_: 15.6). As these initial observations suggested severe sepsis, the patient was taken straight to the operating room.

In the operating room, a laparotomy through the midline incision made earlier revealed a previously unnoticed shrapnel injury in the splenic flexure with necrosis and spillage of phlegmon in the left flank and fasciitis of the fascia and muscles of the abdominal wall and left groin. The patient underwent colectomy with debridement of the necrotic tissues. The bowel was enclosed with nylon and pinned to the abdominal skin. The orthopedic crew then removed the KW fixation and amputated the left palm due to severe necrosis. Afterwards, the patient was admitted to the pediatric intensive care unit (PICU) and was treated with two units each of PC and FFP, broad spectrum antibiotics, and total parenteral nutrition. Repeated blood tests revealed a rise of HB to 11.47 and normalization of WBC and HCO_3_ levels with an albumin level of 2.28. A day after surgery, a second-look laparotomy showed no signs of peritonitis. He underwent peritoneal lavage, and a temporary colostomy from the proximal end of the transverse colon was created. The left groin was closed with a Dexon mesh.

Three days later, a relaparotomy showed that the bowel tissue had a normal color, and fibrin was present. We decided to close the left abdominal wall with a 15∗20-cm Permacol mesh (West Yorkshire, Great Britain). Because the child's family are religious Muslims and Permacol is derived from porcine collagen, the family was given a thorough explanation of the procedure. After receiving their consent, the left abdominal wall was sutured with a 15∗20-cm Permacol mesh in a sublay technique with prolene. Two Jackson Pratt drainages were inserted, one above the mesh and the other in the left flank. The patient was gradually extubated, the chest and Jackson Pratt drains were removed, and he began oral nutrition.

After a week, he underwent three plastic reconstruction surgeries for insertion of a tensor fascia lata flap (TFLF), a musculocutaneous flap to cover the left groin area where the patient had massive skin loss. A month after the injury, with both the abdomen and left groin injuries fully closed, he was discharged, and he was able to return home to Gaza, fully ambulatory with no need of assistance.

Despite the complex political situation, we were able to bring the boy back to our center for a mandatory follow-up examination. After three months, the boy is pleased with the cosmetic outcome in his abdominal area. Furthermore, he has no complaints of abdominal pain, and a physical examination showed no evidence of a hernia.

A combination of rapid assessment and the teamwork of traumatology surgeons, general surgeons, pediatric surgeons, orthopedic surgeons, plastic surgeons, and intensive care physicians and their decision to use the Permacol mesh were the basis of the successful treatment that actually saved the young boy's life.

## 3. Discussion

Permacol is a biomaterial in use since 1980 in a variety of surgical specialties for urological, plastic, and gynaecological procedures [9]. It is a porcine derived acellular dermal sheet composed predominately of type I collagen (93–95%). During the manufacturing process, the cellular components are removed, and the collagen of the dermis is treated with hexamethylene diisocyanate (HMDI) to increase the degree of cross-linking [10].

Trauma patients suffering from abdominal wall defect are likely to have contaminated surgical fields. In this scenario, the question of the preferable technique for abdominal reconstruction arises. Despite the theoretical advantage of Permacol, that is, it allows neovascularization and tissue ingrowth, thereby enabling antibiotic permeation [11, 12], clinical studies investigating its efficacy show conflicting data. On the one hand, some case series report an obvious advantage to using the porcine mesh in contaminated fields. For example, Loganathan et al. [13] reported a series of 15 patients who had repairs of complex hernias, including 10 incisional and four parastomal hernias, some of which had an infected surgical field. Two recurrences occurred, one in a parastomal hernia within 30 days, most likely due to surgical technique, and the other in an incisional hernia, and although it was not clearly stated in the paper, this case was likely a late recurrence. Catena et al. [14] reported a series of seven patients with complicated incisional hernias in contaminated fields. In a mean follow-up of 11 months, no recurrence occurred. Likewise, a series of nine patients by Parker et al. [15], 56% of whom had contaminated wounds (classes II, III, and IV), described only one recurrence in a median follow-up of 18.2 months. On the other hand, recent in vitro and in vivo study [16], comparing three collagen bioprostheses (Collamend, Surgisis, and Permacol) to a control polytetrafluoroethylene mesh in a contaminated field, reported that the collagen bioprostheses failed to show any bacterial adhesion or bacterial clearance benefits. In addition, Rosen et al. [17] reported five years of experience with the repair of infected and contaminated abdominal wall defects utilizing biologic mesh. No long-term infectious complications related to the biologic mesh were reported. However, the long-term durability was less favorable, as over 50% of patients had recurrent hernias within three years. For clarification, they used other biological meshes (Strattice, Alloderm, Biodesign, Xenmatrix, and BioA) and not Permacol, as was used in our case.

On the basis of those studies, we decided that the best technique for reconstruction of the abdominal wall in the eight-year-old trauma patient is with the Permacol mesh.

The use of Permacol in pediatric surgery has been reported for the repair of congenital diaphragmatic hernias [18]. Recently, a small case series [19] described the use of Permacol in pediatric chest wall reconstructions with good short-term results. Concerning abdominal wall reconstruction, Richards et al. [20] and Pentlow et al. [21] described the use of Permacol in pediatric renal transplant recipients with donor size discrepancies. In all cases, primary closure was achieved.

Current studies similar to our case discussing the use of Permacol in a contaminated surgical field concern adult patients undergoing elective or semielective surgery and the use of the mesh in pediatric surgery not related to trauma or with contamination. We describe a pediatric trauma patient who had an abdominal wall reconstruction with Permacol in the presence of a contaminated surgical field. At the end of a three-month follow-up period, no hernia or long-term infectious complications were observed.

## 4. Conclusion

We conclude that Permacol mesh should be considered by pediatric trauma surgeons as a viable alternative to primary closure or synthetic mesh in cases of trauma with contaminated surgical fields.
